# An Update on Current Therapeutic Options in IgA Nephropathy

**DOI:** 10.3390/jcm13040947

**Published:** 2024-02-07

**Authors:** Regina Shaoying Lim, See Cheng Yeo, Jonathan Barratt, Dana V. Rizk

**Affiliations:** 1 Department of Renal Medicine, Tan Tock Seng Hospital, 11 Jalan Tan Tock Seng, Singapore 308433, Singapore; regina_lim@ttsh.com.sg (R.S.L.); see_cheng_yeo@ttsh.com.sg (S.C.Y.); 2 Department of Cardiovascular Sciences, University of Leicester, Leicester LE1 7RH, UK; jb81@leicester.ac.uk; 3 John Walls Renal Unit, University Hospitals of Leicester NHS Trust, Leicester LE5 4PW, UK; 4 Division of Nephrology, Department of Medicine, University of Alabama at Birmingham, ZRB 614, 1720 2nd Avenue South, Birmingham, AL 35294, USA

**Keywords:** IgA, IgA nephropathy, glomerular diseases, clinical trials, treatment, therapy

## Abstract

Immunoglobulin A nephropathy (IgAN) remains the leading cause of primary glomerular disease worldwide. Outcomes are poor with high rates of progressive chronic kidney disease and kidney failure, which contributes to global healthcare costs. Although this disease entity has been described, there were no disease-specific treatments until recently, with the current standard of care focusing on optimal supportive measures including lifestyle modifications and optimization of the renin-angiotensin-aldosterone blockade. However, with significant advances in the understanding of the pathogenesis of IgAN in the past decade, and the acceptance of surrogate outcomes for accelerated drug approval, there have been many new investigational agents tested to target this disease. As these agents become available, we envision a multi-pronged treatment strategy that simultaneously targets the consequences of ongoing nephron loss, stopping any glomerular inflammation, inhibiting pro-fibrotic signals in the glomerulus and tubulo-interstitium, and inhibiting the production of pathogenic IgA molecules. This review is an update on a previous review published in 2021, and we aim to summarize the developments and updates in therapeutic strategies in IgAN and highlight the promising discoveries that are likely to add to our armamentarium.

## 1. Introduction

Immunoglobulin A nephropathy (IgAN) remains the most common cause of primary glomerular disease worldwide [1]. There is significant heterogeneity in clinical presentation, as well as ethnic variability in disease prevalence, clinical course, and response to immunosuppression [2], but overall, it is often a progressive disease with no established disease specific treatment. Current management focuses on optimal supportive care, however, up to 30% of patients still develop kidney failure within 20 years of diagnosis [3,4,5,6], and a more recent report suggests that few patients avoid kidney failure in their lifetime [7]. This confers a significant healthcare burden and cost. 

**Figure 1:**
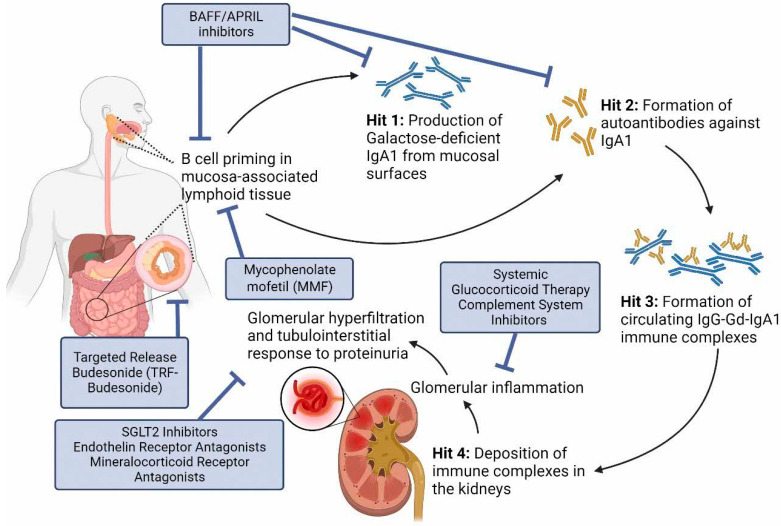
Proposed four-hit hypothesis of IgAN pathogenesis and current therapeutic options. (1) B cell priming and activation in the mucosa-associated lymphoid tissue including Peyer’s patches concentrated in the terminal ileum, resulting in the production of Galactose-deficient IgA1 (Gd-IgA1). (2) Formation of autoantibodies against IgA1 (anti-Gd-IgA1 antibodies). (3) Formation of circulating immune complexes as IgG anti-IgA1 antibodies bind to the hinge region of Gd-IgA1. (4) Deposition of circulating immune complexes in the mesangium through mesangial trapping, which triggers downstream complement activation, tissue injury, and damage. BAFF: B Cell Activating Factor; APRIL: A Proliferation-inducing Ligand; SGLT2 inhibitors: Sodium-glucose cotransporter-2 inhibitors.

New developments in the understanding of the pathogenesis of IgAN as an immune mediated disease has resulted in a rapid expansion of clinical trials testing new therapeutic strategies to target disease-specific pathways. The four-hit hypothesis of IgAN describes 4 sequential steps in disease development: the increased serum levels of galactose-deficient IgA1 (Gd-IgA1) (hit 1), the induction of autoantibodies against Gd-IgA1 (hit 2), formation of circulating immune complexes (hit 3), and their deposition in the kidneys with subsequent inflammation and damage, ultimately leading to decline in kidney function (hit 4), all of which represent potential specific therapeutic targets (Figure 1). The extent of proteinuria has also been identified as a reliable predictor of clinical outcome [8] and a reduction in proteinuria with treatment has now been accepted by regulatory agencies, including the United States Food and Drug Administration (FDA), as a reasonable likely surrogate endpoint predictive of a drug’s longer-term effects on rate of loss of kidney function. The consequence of this change in the regulatory approval process in IgAN has led to an explosion in clinical trials in recent years (Table 1) and the first accelerated approvals of therapies for the treatment of IgAN. 

**Figure 2:**
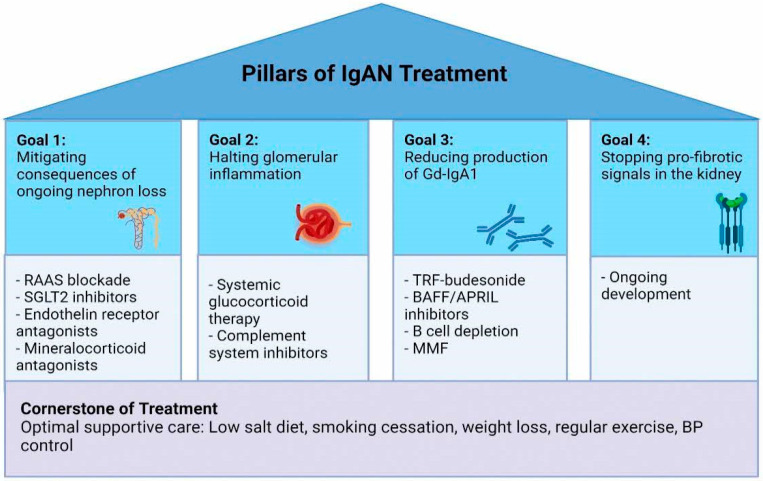
Pillars of IgAN Treatment. The management of patients with IgAN requires a multi-pronged approach to target the predominant pathogenic pathways specific to each patient at each disease timepoint, on top of a cornerstone of optimal supportive care. (1) Mitigation of the consequences of ongoing nephron loss through optimal RAAS blockade, SGLT2 inhibitors, endothelin receptor antagonists, and mineralocorticoid antagonists. (2) Halting glomerular inflammation through the use of systemic glucocorticoid therapy has been long studied for use in IgAN but offers short-term efficacy and comes with significant side effects. With a better understanding of IgAN being an immune mediated disease with activation of the alternative and lectin pathways mediating inflammation, complement pathway inhibitors may be an alternative to limit glomerular inflammation and injury in IgAN. (3) B cells are central to the pathogenesis of IgAN through the production of pathogenic Gd-IgA1. The use of TRF-budesonide can inhibit mucosal IgA production within Peyer’s patches with reduced systemic side effects of glucocorticoid therapy. B cell modulating therapies that inhibit BAFF and APRIL to reduce B cell proliferation and survival, as well as B cell depleting therapies such as borteozomib or felzartamab, may be useful in targeting B cell dysregulation. (4) There are no approved therapies for stopping pro-fibrotic signals in the kidney on the horizon, but we are hopeful for new therapeutic strategies to develop in the future. Gd-IgA1: Galactose-deficient IgA1; SGLT2 inhibitors: RAAS: renin-angiotensin-aldosterone system; Sodium-glucose cotransporter-2 inhibitors; TRF-budesonide: Targeted Release Formulation of Budesonide; BAFF: B Cell Activating Factor; APRIL: A Proliferation-inducing Ligand; MMF: Mycophenolate Mofetil; BP: blood pressure.

Managing patients with IgAN requires a multi-pronged approach that involves mitigating the consequences of ongoing nephron loss, halting glomerular inflammation early, switching off the production of pathogenic IgA, as well as stopping pro-fibrotic signals in the tubulo-interstitium and glomerulus. No single drug can achieve all of these goals, and hence in the future, we will have to look at combining several therapies to prevent kidney failure in patients with IgAN (Figure 2). 

This review highlights the newly approved therapies and ongoing promising clinical trials that may add new drugs to our armamentarium in a constantly evolving landscape in primary IgAN. The treatment of variant forms of IgAN, such as IgA deposition with minimal change disease or rapidly progressive glomerulonephritis, IgA vasculitis, and secondary forms of IgAN is beyond the scope of this review.

## 2. Cornerstone of Treatment: Optimal Supportive Care

Despite advances in our understanding of the pathogenesis of IgAN, no disease-specific treatment was available until recently, and treatment focused on optimal supportive care, with a significant proportion of patients remaining at high risk of chronic kidney disease (CKD) progression. Registry data has shown that a time-averaged proteinuria of >1.0g/day is associated with a significant risk of CKD progression in IgAN [9], and more recent data suggests that even patients traditionally regarded as being low risk (proteinuria < 0.88 g/g or 100 mg/mmol) had high rates of kidney failure within 10 years, indicating the significant risk of CKD progression [7].

Optimal supportive care includes a low salt diet, hyperlipidemia management, smoking cessation, weight loss, regular exercise, blood pressure (BP) control, and renin-angiotensin-aldosterone system (RAAS) blockade with maximally tolerated angiotensin-converting enzyme inhibitor (ACEi) or angiotensin II receptor blocker (ARB) to minimize proteinuria. The Supportive versus immunosuppression Therapy for the treatment of Progressive IgA Nephropathy (STOP-IgAN) trial showed the importance of optimal conservative management when a third of patients who were initially thought to be eligible for randomization, were no longer eligible for the trial after achieving goal proteinuria after a 6-month run-in period of intensified supportive care [10].

The recently published Kidney Disease: Improving Global Outcomes (KDIGO) Clinical Practice Guideline for the Management of Glomerular Diseases 2021 [11] suggests a stricter systolic BP control target of <120 mmHg using standardized office BP measurement, considering evidence that more intensive BP control is protective for cardiovascular outcomes [12]. However, this target has not been validated in glomerular diseases or specifically in IgAN. 

Despite optimal supportive care, a significant residual risk of CKD progression persists and outcomes remain poor. Long-term follow-up of the patients from the STOP-IgAN trial showed that almost half of the patients reached the primary composite end point of all-cause mortality, kidney failure, and a 40% decline in glomerular filtration rate (GFR) after 7.4 years of follow up [13]. Hence, there is a need to consider additional treatment options for this group of patients. 

## 3. Further Mitigation of Ongoing Nephron Loss

### 3.1. Sodium-Glucose Cotransporter-2 (SGLT2) Inhibitors

Multiple large trials have demonstrated the kidney protective effects of sodium-glucose cotransporter-2 (SGLT2) inhibitors in patients with kidney diseases beyond diabetic nephropathy, thought to be mediated primarily through natriuresis, reduction in intraglomerular pressure, blood pressure reduction, and/or weight loss. It is also postulated that cellular and metabolic effects of SGLT2 inhibitors may also explain some of their kidney protective effects, although the role of these mechanisms in IgAN is less certain and further studies are needed.

The Dapagliflozin and Prevention of Adverse Outcomes in Chronic Kidney Disease (DAPA-CKD) trial assessed the efficacy and safety of dapagliflozin in patients with or without type 2 diabetes [14]. The study included 270 patients with IgAN and was prematurely terminated at 2.4 years due to efficacy, with the dapagliflozin group reaching the primary composite outcome of a 50% decline in GFR, kidney failure, or death from renal, or cardiovascular causes less frequently than the placebo group (HR 0.56, 95% CI 0.45–0.68). The effects of dapagliflozin were similar in patients with or without type 2 diabetes. A prespecified subgroup analysis of the 270 patients with IgAN confirmed similar favorable results of GFR loss and proteinuria in the dapagliflozin arm [15]. 

The Study of Heart and Kidney Protection with Empagliflozin (EMPA-KIDNEY trial) also recruited patients with or without diabetes who had CKD, and included 817 patients with IgAN [16]. Patients were randomized to empagliflozin or placebo, and the trial was terminated early at 2.0 years due to efficacy in the empagliflozin group. 

The primary composite outcome of progression of kidney disease or death from cardiovascular cause occurred less frequently in the empagliflozin group compared to placebo (HR 0.72, 95% CI 0.64–0.82), which was consistent regardless of diabetic status or GFR range. 

The SGLT2 Inhibitor Meta-Analysis Cardio-Renal Trialists’ Consortium (SMART-C) conducted a meta-analysis of 13 randomized controlled trials to provide pooled estimates of the effect of SGLT2 inhibitors [17]. This analysis found that SGLT2 inhibitors reduced the risk of kidney disease progression by 40% (relative risk 0.60, 95% CI 0.46–0.78) in patients with glomerular diseases, which was similar between disease subcategories of IgAN, focal segmental glomerulosclerosis (FSGS) and other glomerulopathies. Specifically, in IgAN, SGLT2 inhibitors reduced the risk of kidney disease progression by 51% (relative risk 0.49, 95% CI 0.32–0.74). 

### 3.2. Endothelin Receptor Antagonists

Endothelin-1 (ET-1) has been shown to contribute to IgAN pathogenesis through activation of endothelin A (ETA) receptors, leading to vasoconstriction, podocyte injury, inflammation, and fibrosis which lead to the progression of chronic kidney disease [18]. 

Sparsentan is a dual antagonist of angiotensin and endothelin receptors that has been shown in a phase II study to reduce proteinuria compared to irbesartan alone in patients with focal segmental glomerulosclerosis (FSGS) [19]. The phase III PROTECT trial randomized patients with biopsy-proven IgAN and persistent proteinuria > 1.0 g/day despite at least 12 weeks of best supportive care to sparsentan 400 mg once daily or irbesartan 300 mg once daily. A prespecified interim analysis after 36 weeks showed a greater reduction of proteinuria in the sparsentan group (−49.8%) compared to placebo (−15.1%) which was independent of blood pressure lowering effect, with a similar side effect profile between groups [20]. This led the FDA to grant accelerated approval to sparsentan for IgAN treatment in February 2023. A two-year follow-up analysis confirmed the sustained beneficial effects of sparsentan [21]. Proteinuria was 40% lower in the sparsentan group at 110 weeks compared to irbesartan, and patients in the sparsentan group also had a slower rate of eGFR decline compared to irbesartan (−2.7 mL/min/1.73 m^2^ compared to −3.8 mL/min/1.73 m^2^), with no significant differences in adverse events between the groups [21]. 

Another phase III trial evaluating the efficacy of atrasentan, a selective ETA receptor antagonist, is currently ongoing (ClinicalTrials.gov Identifier: NCT04573478). Topline results from the interim analysis were recently announced, with atrasentan demonstrating superiority over placebo in the reduction of proteinuria in IgAN patients [22]. 

### 3.3. Mineralocorticoid Receptor Antagonists (MRA)

It is also postulated that blocking mineralocorticoid receptors expressed on podocytes, endothelial, and mesangial cells prevents glomerular and tubulointerstitial fibrosis and slow kidney disease progression [23]. Traditional MRAs, such as spironolactone and eplerenone, when used together with ACEi or ARB treatment, significantly reduce proteinuria but increase the risk of hyperkalemia, with uncertain long-term impact on kidney outcomes [24]. Finerenone is a novel, nonsteroidal, selective MRA that reduces the risks of CKD progression and cardiovascular events in patients with CKD and type 2 diabetes [25]. The FIND-CKD trial is currently underway to evaluate the effect of finerenone in the non-diabetic CKD patient population (ClinicalTrials.gov Identifier: NCT05047263), including individuals with IgAN. 

## 4. Halting Glomerular Inflammation

### 4.1. The Role of Systemic Glucocorticoid Therapy

Glucocorticoid therapy has been used in IgAN for many years. Earlier non-randomized and randomized single center trials had shown their potential for reducing proteinuria and delaying CKD progression in high-risk patients with proteinuria > 1 g/day [26,27,28,29,30,31], however, these trials were not conducted in an era where supportive care was well established, and the use of RAAS blockade was also inconsistent. The trials also did not collect steroid-related serious adverse events systematically. 

The STOP-IgAN and the Therapeutic Evaluation of Steroids in IgA Nephropathy Global (TESTING) studies mandated a run-in period to optimize supportive care, subsequently recruiting patients with significant residual proteinuria. The STOP-IgAN trial was conducted in a European population, and patients who had persistent proteinuria > 0.75 g/day despite the run-in phase were randomized to immunosuppressive therapy (corticosteroids if GFR > 60 mL/min or cyclophosphamide followed by azathioprine and corticosteroids if GFR was 30–59 mL/min) versus supportive care alone [10]. At 3 years, although the addition of immunosuppression resulted in a reduction in proteinuria compared to supportive care alone, there was no difference in GFR decline between both groups, and more adverse events were observed in the immunosuppression group. Longer-term (10-year) observational data from the STOP-IgAN cohort confirmed no difference in renal outcomes between the two groups [13]. 

The TESTING trial included a majority Chinese population with more severe disease (compared to the STOP-IgAN cohort) with higher median proteinuria and annual GFR decline in the placebo group. TESTING randomized patients who had persistent proteinuria of >1 g/day after a 3-month run-in period to methylprednisolone 0.6–0.8 mg/kg/day versus placebo [32]. It was halted after 2.1 years of follow-up with 262 patients recruited, due to excessive adverse events in the corticosteroid group including 2 deaths related to infection. The preliminary analysis showed a signal favoring a lower event rate of kidney failure, death due to kidney failure, or a 40% decline in GFR in the steroid group. TESTING was resumed with a steroid dose reduction (methylprednisolone 0.4 mg/kg/day), the addition of trimethoprim/sulfamethoxazole prophylaxis for *Pneumocystis jiroveci* pneumonia, and raising the lower limit of eGFR range to 30 mL/min/1.73 m^2^. An additional 241 patients were recruited [33]. After 4.2 years of follow-up, the primary outcome of kidney failure, death due to kidney failure, or a 40% decline in GFR occurred less commonly in both the full-dose (HR 0.58, 95% CI 0.41–0.81) and reduced-dose methylprednisolone groups (HR 0.27, 95% CI 0.11–0.65) compared to placebo. However, serious adverse events occurred more frequently in both methylprednisolone groups compared to placebo, primarily with full-dose therapy. Interestingly, proteinuria was significantly lower in the corticosteroid treatment groups, but the reduction was mostly seen relatively early and was no longer apparent by 3 years of follow-up, consistent with a short-term anti-inflammatory effect, and it remains unclear what this means in terms of longer-term outcomes or the need for repeated courses of treatment. 

Although studies have reported the short-term efficacy of steroid therapy in patients at high risk of progressive disease, systemic glucocorticoid therapy has also been shown to be associated with significant adverse events in all trials alongside significant patient-reported intolerability [34]. Currently, the 2021 KDIGO Clinical Practice Guideline suggests that patients at high risk of progressive loss of kidney function are first offered the opportunity of enrolment in a clinical trial. If this is not possible, and only after a thorough toxicity risk stratification, some patients may be considered for a 6-month course of glucocorticoid therapy (Grade 2B). Importantly, systemic glucocorticoids should be given with extreme caution or avoided entirely in patients at high risk of adverse effects [11].

### 4.2. The Complement System and IgAN

There is increasing evidence for a major role of complement activation in driving both glomerular and tubulointerstitial inflammation in IgAN. Multiple complement proteins are found co-deposited with IgA immune complexes including C3, properdin, C4d, mannose-binding lectin as well as terminal complement components in IgAN [35,36,37,38,39]. However, C1q is absent in the majority of IgAN biopsy specimens [35]. These findings suggest that complement activation via the alternative and lectin pathways, and not the classical pathway, is the triggering event for complement activation within the glomerulus in IgAN. Several genome-wide association (GWA) studies have shown an association between complement genes and the risk of developing IgAN, including a deletion of the complement regulatory genes complement factor H-related genes 1 and 3 (CFHR1 and CFHR3), which is protective and reduces the risk of developing IgAN [40]. 

#### 4.2.1. Lectin Pathway Inhibition as an Anti-Inflammatory Approach in IgAN

Mannose-binding lectin-associated serine protease 2 (MASP-2) is a component of the lectin pathway that triggers C3 convertase formation. Narsoplimab (OMS721) is a monoclonal antibody that selectively inhibits MASP-2 and is administered via weekly infusions. A phase II trial with 2 sub-studies, one with IgAN patients who received corticosteroids and another without, demonstrated its efficacy at 18 weeks in proteinuria reduction and suggested that narsoplimab is safe and well tolerated [41]. This led to the launch of a phase III study, ARTEMIS-IgAN (ClinicalTrials.gov Identifier: NCT03608033). Late in 2023, ARTEMIS-IgAN was terminated early after the pre-specified interim analysis did not demonstrate a significant proteinuria reduction with narsoplimab [42]. 

#### 4.2.2. Alternative Pathway Inhibition as an Anti-Inflammatory Approach in IgAN

The alternative complement pathway is a constitutively active part of the immune system which plays a role in the amplification of the classical and lectin pathways. Two key proteins of the alternative pathway are Factor B and Factor D, and drugs inhibiting both proteins are currently being evaluated in IgAN as a means of limiting glomerular inflammation driven by alternative pathway activation. 

Iptacopan (LNP023) is a potent oral selective inhibitor of factor B. Its efficacy was demonstrated in a phase II study of 66 patients with biopsy-proven IgAN, which showed a dose-response effect of iptacopan at 3 and 6 months versus placebo [43]. Proteinuria was reduced in the iptacopan 200 mg twice daily group by up to 40% (95% CI 16–53%) compared to placebo, with no serious treatment-related adverse events reported [43]. A phase III trial evaluating the efficacy and safety of iptacopan is currently recruiting (APPLAUSE-IgAN, ClinicalTrials.gov Identifier: NCT04578834). A pre-specified interim analysis demonstrated the superiority of iptacopan over placebo in proteinuria reduction at 9 months [22]. 

IONIS FB-LRx is a subcutaneously administered investigational factor B antisense oligonucleotide that inhibits factor B production. Preliminary results from an open-label phase II study were presented at the American Society of Nephrology (ASN) Kidney Week in 2022. Ten patients with IgAN treated with IONIS-FB-LRx had a 44% reduction in proteinuria at week 29 (ClinicalTrials.gov Identifier: NCT04014335). Based on these results, the phase III Imagination trial has started recruitment (ClinicalTrials.gov Identifier: NCT05797610). 

Vemircopan (ALXN2050) is an oral active complement factor D inhibitor currently being studied in a Phase II study in IgAN and proliferative lupus nephritis (ClinicalTrials.gov Identifier: NCT05097989). 

#### 4.2.3. Terminal Complement Pathway Inhibitors

Pegcetacoplan (APL-2), a C3 and C3b inhibitor, has been evaluated in a phase II clinical trial as a treatment option for IgAN, lupus nephritis, primary membranous nephropathy (MN), and C3 glomerulopathy (ClinicalTrials.gov Identifier: NCT03453619), however, no data are available at this time. ARO-C3 is an investigational RNA interference agent that reduces hepatic production of C3 and is being evaluated for safety in a phase I trial (ClinicalTrials.gov Identifier: NCT05083364) which is currently recruiting. Interim results showed a dose-dependent reduction in serum C3 and AH50, a marker of alternative complement activity, with no serious adverse events reported. 

It will move forward to phase II studies on participants with complement-mediated diseases including C3 glomerulopathy and IgAN [44]. 

C5a is a strong chemoattractant that recruits immune cells into sites of inflammation. Avacopan (CCX168), a selective C5a receptor antagonist that can dampen the complement-mediated inflammatory response, has shown benefit in the treatment of anti-neutrophil cytoplasmic antibody-associated vasculitis [45,46]. As it does not affect C5b-9 production, it is also thought to be associated with a lower risk of infections by encapsulated organisms such as Neisserial species. A pilot open-label phase II trial of avacopan in 7 IgAN patients with proteinuria > 1 g/day and eGFR > 60 mL/min or eGFR > 45 mL/min if GFR had not declined significantly over the preceding 24 weeks was conducted [47]. At the end of 12 weeks, 6 out of 7 patients had an improvement in proteinuria during the treatment period with avacopan, and 3 of these patients had a numerical improvement of proteinuria of about 50%. Larger randomized trials with a longer duration of follow-up are required to confirm the efficacy and safety of C5a inhibition in IgAN. 

Cemdisiran (ALN-CC5) is a small interfering RNA that blocks terminal complement pathway activation and consequent inflammation and tissue injury by suppressing C5 production in the liver. Results from a phase II study were presented in 2022 at the ASN Kidney Week (ClinicalTrials.gov, Identifier: NCT03841448). Thirty-one patients with IgAN and proteinuria > 1 g/day were randomized 2:1 to subcutaneous cemdisiran vs. placebo, which resulted in a 37.4% mean reduction in proteinuria and stabilization of GFR in favor of cemdisiran at week 32. It is unclear if there will be a phase III study of cemdisiran in IgAN. 

Ravulizumab is a monoclonal antibody against C5 that is currently being investigated in a phase II clinical trial for IgAN (ClinicalTrials.gov Identifier: NCT04564339), and primary analysis results were recently presented in 2023 at the ASN Kidney Week. Sixty-six patients with IgAN were randomized 2:1 to ravulizumab vs. placebo, and proteinuria reduction was greater in the ravulizumab groups at 26 weeks (40.3% vs. 10.9%) while GFR remained stable. 

## 5. Reducing Production of Pathogenic IgA

### 5.1. Targeted Release Formulation of Budesonide (TRF-Budesonide)

The association between mucosal inflammation and IgAN is well established. Clinical observation of synpharyngitic hematuria in patients with IgAN has long been described, and more recently, genetic variants related to mucosal immunity and systemic production of mucosal-type IgA1 in IgAN have been shown to increase disease risk [48]. Mucosal-associated lymphoid tissue, particularly the Peyer’s patches, which are concentrated in the distal ileum, are involved in the synthesis of Gd-IgA1 which is the first step of the 4-hit hypothesis in IgAN pathogenesis. Targeted release formulation of budesonide (TRF-budesonide), or Nefecon, is an oral targeted release formulation of corticosteroids which delays release of the drug until it reaches the distal ileum, and hence reduces the production of Gd-IgA1 [49]. This has been thought to improve treatment efficacy at the site of action and reduce systemic glucocorticoid toxicity because of Nefecon’s high first-pass effect. 

The NefIgArd trial was a phase III multicenter randomized controlled trial testing the efficacy and safety of Nefecon 16 mg/day compared to placebo over 9 months in patients with IgAN at high risk of progressive renal failure as defined by proteinuria of >1 g/day despite optimized supportive care for at least 3 months, with further follow up to 2 years. At 9 months, proteinuria was 27% lower in the Nefecon group compared with placebo [50]. At 2 year follow up study showed a treatment benefit with Nefecon over placebo in terms of time-weighted average of GFR, with a GFR change of −2.47 mL/min/1.73 m^2^ in the Nefecon group compared to −7.52 mL/min/1.73 m^2^ in placebo [51]. Nefecon was also generally well tolerated with a more acceptable safety profile compared to systemic glucocorticoid therapy.

Nefecon was granted accelerated approval by the United States FDA in December 2021 and full approval for treatment of IgAN in December 2023.

### 5.2. Targeting B-Cell Dysregulation through BAFF/APRIL Inhibition

There is increasing evidence that T cell-independent B cell activation, mediated by B-Cell Activating Factor (BAFF) and A Proliferation Inducing Ligand (APRIL), is responsible for the production of Gd-IgA1 at mucosal surfaces [52]. Dysregulation of this pathway could provide an explanation for the occurrence of synpharyngitic hematuria in patients with IgAN, where a mucosal infection triggers increased mucosal Gd-IgA1 production and hence a disease flare, and the chronic elevation of serum Gd-IgA1 levels in IgAN [53,54]. 

B cell maturation and survival is dependent on BAFF and APRIL. Transgenic mice with BAFF overexpression develop an IgA-mediated nephritis, while mice who overexpress BAFF but are IgA deficient do not develop this phenotype [55]. APRIL also shares some of the same B cell signaling receptors. Elevated plasma APRIL levels have been documented in patients with IgAN and correlated with higher Gd-IgA1 levels as well as a more severe clinical phenotype [56]. It has also been postulated that APRIL controls immunoglobulin class switch recombination to IgA1. Additionally, GWA studies have identified *TNFSF13* (which encodes APRIL) as a genome-wide significant risk locus for IgAN [57], and this has been confirmed in the recent large metaGWA study, alongside identification of *TNFSF13B* (encoding the APRIL receptor, Transmembrane activator and CAML interactor, TACI) as another genome-wide significant risk locus [58], confirming the importance of this cytokine ligand-receptor pair in the pathogenesis of IgAN. 

Sibeprenlimab (VIS639) is a humanized IgG2 monoclonal antibody that inhibits APRIL. A phase II multicenter double-blind randomized controlled trial evaluated 12 monthly intravenous infusions of sibeprenlimab at doses of 2, 4, or 8 mg per kilogram body weight vs. placebo in patients with IgAN who were at high risk for disease progression [59]. At 12 months, the sibeprenlimab groups demonstrated greater reductions in 24-h urinary protein-to-creatinine ratio from baseline (47.2 ± 8.2%, 58.8 ± 6.1%, 62.0 ± 5.7% in the 2 mg, 4 mg, and 8 mg groups respectively) compared to placebo (20.0 ± 12.6%) [59]. Phase III and open-label extension studies are currently ongoing (ClinicalTrials.gov Identifier: NCT05248646 and NCT05248659). 

BION-1301, or Zigakibart, is another novel humanized anti-APRIL monoclonal antibody that has shown promising interim results from phase I and II studies. It is well tolerated in IgAN patients with no serious adverse effects reported. Zigakibart therapy led to a reduction in proteinuria as early as 12 weeks with an associated reduction in Gd-IgA1 levels [60]. A phase III study is currently ongoing (ClinicalTrials.gov Identifier: NCT05852938).

Dual targeting of both BAFF and APRIL with atacicept, a novel B-cell targeted immunomodulator, has also shown a dose-dependent reduction in proteinuria and Gd-IgA1 antibody levels in a phase II study [61]. A phase IIB clinical trial randomized 116 patients with IgAN in a 2:2:1:2 fashion to atacicept 150 mg, 75 mg, 25 mg vs. placebo. The atacicept 150 mg arm was the only individual treatment arm that demonstrated a greater reduction in proteinuria from baseline compared to placebo (41% vs. 10%) at week 24 [62]. A phase III study evaluating atacicept 150 mg vs. placebo is currently ongoing (ClinicalTrials.gov Identifier: NCT04716231). Telitacicept, another fusion protein inhibiting both BAFF and APRIL, has also shown clinical benefit in a phase II randomized controlled trial involving 44 Chinese patients with IgAN and persistent proteinuria > 0.75 g/day despite optimal supportive therapy [63]. Treatment with telitacicept reduced proteinuria compared to placebo, and GFR remained stable over time with no severe adverse effects reported. A phase III trial is ongoing (ClinicalTrials.gov Identifier: NCT05799287). 

Povetacicept (ALPN-303) is another dual BAFF/APRIL antagonist that has also been studied in patients with IgAN, primary MN and lupus nephritis. A phase IB/IIA study (ClinicalTrials.gov Identifier: NCT05732402) involving patients with IgAN and primary MN has started recruitment, and preliminary data presented in 2023 at the ASN Kidney Week suggests that povetacicept is well tolerated and results in early reductions in proteinuria and disease-specific markers in patients with IgAN and primary MN. 

### 5.3. B Cell Depleting Agents

A randomized controlled trial of CD20 depletion in IgAN failed to demonstrate the benefit of adding rituximab on top of standard therapy in proteinuria reduction, stabilization of renal function, or reduction in Gd-IgA1 and anti-Gd-IgA1 antibody levels [64]. An alternative depletion approach currently being evaluated in IgAN adapted from the treatment of the plasma cell malignancy multiple myeloma is the use of CD38 depletion. Unlike B cells, plasma cells display high expression of CD38^+^ while expressing very low-level expression of CD20 [65]. 

Felzartamab (ClinicalTrials.gov Identifier: NCT05065970) and mezagitamab (ClinicalTrials.gov Identifier: NCT05174221) are anti-CD38 antibody therapies currently being evaluated in early phase studies in IgAN. Felzartamab has shown efficacy in preliminary phase I/IIA trials in anti-phospholipase A2 receptor (PLA2R) antibody-positive MN [66].

The proteosome inhibitor borteozomib (Velcade), a plasma cell-depleting agent that has been used in the treatment of multiple myeloma, has been studied in a pilot open-label trial involving 8 patients with IgAN [67]. Three of these 8 patients achieved complete remission after 4 doses of borteozomib at 1-year follow-up, suggesting that plasma cell depletion could potentially improve outcomes in IgAN. However, larger trials are still required to demonstrate efficacy and safety.

### 5.4. Inhibiting Lymphocyte Proliferation with Mycophenolate Mofetil (MMF)

Mycophenolate Mofetil (MMF) is a potent anti-metabolite that inhibits B and T cell proliferation. Data regarding its use in IgAN are conflicting. Trials of MMF performed in mainly Caucasian patients [68,69,70] have not shown benefits concerning improvement in proteinuria or kidney function. However, trials from East Asia have reported benefits. In a study from Hong Kong, treatment with MMF reduced proteinuria in patients with IgAN of Chinese ethnicity [71]. 

The recently published Effectiveness of Mycophenolate Mofetil Among Patients with Progressive IgA Nephropathy (MAIN) trial [72] adds to this growing body of evidence in Chinese patients. MAIN was a randomized non-blinded controlled trial that compared the use of MMF 1.5 g/day vs. placebo for 12 months in 170 Chinese patients who had persistent proteinuria of >0.75 g/day after a 3-month run-in period of optimal supportive care. The primary composite outcome of doubling of serum creatinine, kidney failure, or death due to a renal or cardiovascular cause occurred less frequently in the MMF group compared to placebo (adjusted HR 0.23, 95% CI 0.09–0.63) after 12 months. 157 participants who completed the trial were followed up for a median of 60 months, 80 participants in the MMF group (14 of whom continued MMF throughout the entire follow-up period and 66 who discontinued MMF after the trial phase), and 77 in the placebo group. At the end of this follow-up, the mean annual loss of GFR was lowest in the group that continued MMF throughout the post-trial period (4.1 mL/min/1.73 m^2^) compared to those who were randomized to MMF but stopped during the post-trial phase (6.2 mL/min/1.73 m^2^) and placebo (7.1 mL/min/1.73 m^2^). 

## 6. Stopping Pro-Fibrotic Signals in the Kidney

The upregulation of pro-fibrotic signals in the kidney in IgAN is thought to be mediated by several cytokines and growth factors including platelet-derived growth factor (PDGF) and transforming growth factor β1 (TGF-β1), which stimulate extracellular matrix production and tissue fibrosis. Intra-renal TGF-β1 expression has been shown to correlate with extracellular matrix accumulation and poorer prognostic markers in IgAN [73,74,75]. 

Potential therapeutic interventions targeting inhibition of TGF- β1 have been shown to prevent fibrosis in experimental mice models [74,76], however, this has not translated to clinical benefit in humans and there are no anti-fibrotic drugs on the horizon for use in IgAN at present [77]. 

## 7. Discussion

IgAN is the most common cause of primary glomerular disease and displays extensive clinical heterogeneity across patient populations. Clinical outcomes have remained static over the past few decades. There is an unmet need for disease-specific treatments that can be individualized to address a given patient’s risk of kidney failure, ideally targeting those predominant pathogenic pathways operating at that moment in time and addressing the long-term risk of adverse kidney outcomes arising from immune and non-immune mediated pathways in IgAN. There have been significant advances in our understanding of IgAN as an immune-mediated disease caused by dysregulation of the mucosal and innate immune systems which we have outlined in this review. This improved understanding, alongside a more pragmatic drug approval framework has renewed interest in the development of new therapeutic strategies targeted at specific disease pathways. Consequently, several clinical trials have been conducted in the past few years. We remain hopeful that there will be more drugs approved for the treatment of IgAN soon. With the availability of more drugs clinicians will have to evaluate treatment regimens that may include novel drug combinations or sequential therapies to maximize efficacy, while minimizing toxicity and ensuring patient tolerability. Moving forward, advancements in “omics” technologies are likely to lead to the discovery of new biomarkers that will refine prognostication and ensure we use the *right* drug(s) for the *right* patient at the *right* time in the natural history of their disease. 

## Figures and Tables

**Table 1:** Recent trials in IgAN.

Therapy	Mechanism of Action	Clinical Trial	Primary and Key Secondary Endpoints	Main Results
**1. Mitigating the Consequences of Ongoing Nephron Loss**				
Sparsentan	Dual endothelin A and angiotensin II inhibitor	PROTECT * NCT03762850—completed Phase III randomized double-blind, active-controlled trial	Change in proteinuria at 36 weeks Change in eGFR at 110 weeks	Reduction of proteinuria greater in the sparsentan group (−49.8%) than the irbesartan group (−15.1%) eGFR chronic 2-year slope (weeks 6–110) was −2.7 mL/min per 1.73 m^2^ per year versus −3.8 mL/min per 1.73 m^2^ per year (difference 1.1 mL/min per 1.73 m^2^ per year, 95% CI 0.1 to 2.1; *p* = 0.037); eGFR total 2-year slope (day 1-week 110) was −2.9 mL/min per 1.73 m^2^ per year versus −3.9 mL/min per 1.73 m^2^ per year (difference 1.0 mL/min per 1.73 m^2^ per year, 95% CI −0.03 to 1.94; *p* = 0.058).
Atrasentan	Selective endothelin A receptor inhibitor	ALIGN * NCT04573478—ongoing Phase III randomized double-blind, placebo-controlled trial	Change in proteinuria at 24 weeks Change in eGFR at 2.6 years	Awaited
**2. Halting Glomerular Inflammation**				
Narsoplimab (OMS721)	Blocks lectin pathway activation by inhibiting MASP-2	* NCT02682407—completed Staged phase II study	Change in proteinuria at 12 weeks	Reductions of proteinuria ranges from 54% to 95%, compared to baseline
		ARTERMIS-IgAN * NCT03608033—terminated early Phase III randomized double-blind, placebo-controlled trial	Change in proteinuria at 36 weeks Change in eGFR up to 144 weeks	Trial terminated due to lack of efficacy
Iptacopan (LNP023)	Blocks alternative pathway activation by inhibiting Factor B	* NCT03373461—completed Phase II randomized double-blind trial	Change in proteinuria at 3 months	Reduction of proteinuria of 23% achieved with iptacopan (200 mg twice daily arm), compared to placebo
		APPLAUSE-IgAN * NCT04578834—ongoing Phase III randomized double-blind, placebo-controlled trial	Change in proteinuria at 9 months Change in eGFR at 9 and 24 months	Awaited
IONIS-FB-LRx	Blocks alternative pathway activation by inhibiting Factor B	* NCT04014335—completed Phase II open-label study	Change in proteinuria at 29 weeks	Eight of 10 patients had a reduction in proteinuria at week 29
		IMAGINATION * NCT05797610—ongoing Phase III randomized double-blind placebo-controlled trial	Change in proteinuria at 37 weeks Change in eGFR at 105 weeks	Awaited
Pegcetacoplan (APL-2)	Blocks terminal pathway activation by inhibiting C3	* NCT03453619—active, not recruiting Phase II single-arm open-label trial	Change in proteinuria at 48 weeks	Awaited
ARO-C3	Blocks terminal pathway activation by inhibiting C3	* NCT05083364—ongoing Phase I/II placebo-controlled study	Change in C3 at 169 days	Awaited
Avacopan (CCX168)	C5a receptor inhibitor	* NCT02384317—completed Pilot phase II open-label trial	Change in proteinuria at 12 weeks	Six of seven patients had improvement in the UPCR during the treatment period, three of whom had a improvement of 50%, compared to baseline
Cemdisiran (ALN-CC5)	Blocks terminal pathway activation by inhibiting C5	* NCT03841448—completed Phase II randomized double-blind, placebo-controlled trial	Change in proteinuria at 32 weeks	Reduction (placebo-adjusted) of proteinuria of 37.4%
Raviluzumab	Blocks terminal pathway activation by inhibiting C5	* NCT04564339—ongoing Phase II randomized double-blind, placebo-controlled trial	Change in proteinuria at 26 weeks	Reduction (placebo-adjusted) of proteinuria of 33.1%
Vemircopan (ALXN2050)	Blocks alternative pathway activation by inhibiting Factor D	* NCT05097989—ongoing Phase II randomized double-blind, placebo-controlled trial	Change in proteinuria at 26 weeks	Awaited
**3. Reducing Production of Pathogenic IgA**				
Nefecon (TRF-Budesonide)	Modulates mucosal B cells and plasma cells in the gut associated lymphoid tissue of the small intestine	NEFIGAN * NCT01738035—completed Phase IIB randomized double-blind, placebo-controlled trial	Change in proteinuria at 9 months	Reduction of proteinuria of 24.4% (27.3% in 16 mg treatment group; 21.5% in 8 mg treatment group), compared to placebo
		NefigArd * NCT03643965—completed Phase III randomized double-blind, placebo-controlled trial	Change in proteinuria at 9 months Change in eGFR at 9 and 12 months	Reduction of proteinuria of 27%, compared to placebo; Improvement of eGFR decline by 5.05 mL/min per 1.73 m^2^, compared to placebo
Mycophenolate Mofetil (MMF)	Inhibits lymphocyte proliferation	MAIN * NCT01854814—completed Randomized open-label trial	Composite of doubling of serum creatinine, kidney failure, or death due to kidney disease Progression of chronic kidney disease	77% risk reduction of composite end points (doubling of serum creatinine, kidney failure, or death due to kidney disease)
Sibeprenlimab (VIS639)	Inhibits APRIL	ENVISION * NCT04287985–completed Phase II randomized controlled trial	Change in proteinuria at 12 months	Reduction of proteinuria between 47.2–62.0% in treatment arm compared to 20.0% in placebo arm
		VISIONARY * NCT05248646—ongoing Phase III randomized controlled trial	Change in proteinuria at 9 months Change in eGFR at 24 months	Awaited
Zigakibart (BION-1301)	Inhibits APRIL	* NCT03945318—interim analysis, awaiting final results Phase I and phase II open-label extension	Change in proteinuria at 3 months	Reduction of proteinuria of 30.4% at 12 weeks, compared to baseline; reduction of proteinuria of 66.9% at 52 weeks, compared to baseline
		BEYOND * NCT05852938—ongoing Phase III randomized double-blind, placebo-controlled trial	Change in proteinuria at 40 weeks Change in eGFR at 104 weeks	Awaited
Atacicept	Recombinant fusion protein that binds BAFF and APRIL	JANUS * NCT02808429—completed Phase II randomized controlled trial	Change in proteinuria at 24, 48 and 72 weeks	Reduction of proteinuria of 24% at 12 weeks, 38% at 48 weeks and 50% at 72 weeks with atacicept 25 mg, compared to baseline
		**ORIGIN3** (Phase 2b) * NCT04716231—completed Phase IIb randomized double-blind, placebo-controlled trial	Change in proteinuria at 24 weeks Change in eGFR at 24 weeks	Reduction of proteinuria of 33%, compared to placebo group
		**ORIGIN3** (Phase 3) * NCT04716231-ongoing Phase III randomized controlled trial	Change in proteinuria at 36 weeks Change in eGFR at 104 weeks	Awaited
Telitacicept	Inhibits BAFF and APRIL	* NCT04291781—completed Phase II randomized placebo-controlled trial	Change in proteinuria at 24 weeks	Reduction of proteinuria of 25–49%, compared to baseline
		* NCT05799287—ongoing Phase III randomized double-blind, placebo-controlled trial	Change in proteinuria at 104 weeks Change in eGFR at 3 years	Awaited
Povetacicept (ALPN-303)	Inhibits BAFF and APRIL	* NCT05732402—ongoing Phase I/II open-label trial	Change in proteinuria	Awaited
Borteozomib (Velcade)	Plasma cell depletion	* NCT01103778—completed Pilot open-label trial	Change in proteinuria at 1 year Change in eGFR at 1 year	Three of eight patients had full remission defined as proteinuria of less than 300 mg per day
Felzartamab	Plasma cell depletion	NCT05065970—ongoing Phase II randomized placebo-controlled trial	Change in proteinuria	Awaited
Mezagitamab	Plasma cell depletion	* NCT05174221—ongoing Phase IB open-label study	Change in proteinuria at 36 weeks	Awaited

eGFR: estimated glomerular filtration rate; APRIL: A Proliferation-inducing Ligand; MASP-2: Mannan-binding lectin-associated serine protease-2. * shows ClinicalTrials.gov Identifier. Data from www.clinicaltrials.gov (accessed on 30 January 2024).

## Data Availability

Not applicable.
